# ‘We must treat them like all the other people’: Evaluating the Integrated Key Populations Sensitivity Training Programme for Healthcare Workers in South Africa

**DOI:** 10.4102/sajhivmed.v20i1.909

**Published:** 2019-04-30

**Authors:** Zoe Duby, Francisco Fong-Jaen, Busisiwe Nkosi, Benjamin Brown, Andrew Scheibe

**Affiliations:** 1 Division of Social and Behavioural Sciences, School of Public Health and Family Medicine, University of Cape Town, Cape Town, South Africa; 2 Desmond Tutu HIV Centre, Department of Medicine, University of Cape Town, Cape Town, South Africa; 3 School of Public Health and Family Medicine, University of Cape Town, Cape Town, South Africa

**Keywords:** Men who have sex with men, Sex workers, People who use drugs, Sensitisation Training, Healthcare workers, South Africa

## Abstract

**Background:**

Sensitisation training can reduce judgemental and discriminatory attitudes amongst healthcare workers. The ‘Integrated Key Populations Sensitivity Training Programme for Healthcare Workers in South Africa’ aimed to improve access to appropriate and non-judgemental health services for ‘key populations’, specifically men who have sex with men, sex workers and people who use drugs, through the sensitisation of healthcare workers.

**Objectives:**

The aim of this study was to evaluate the effects of the integrated key population sensitisation training intervention for healthcare workers, conducted between 2013 and 2014 in South Africa.

**Methods:**

This study used a combination of qualitative and quantitative methods. Qualitative methods compared attitudes between healthcare workers who received the training intervention and those who did not. Quantitative methods were used to compare similar changes in awareness amongst healthcare workers before and after receiving the training. We explored shifts in attitudes towards key populations, changes in awareness of health issues related to stigma, discrimination, and changes in capacity to manage sexual health and HIV risk behaviours, including substance use and anal sex.

**Results:**

The findings indicate that the training intervention resulted in a shift in attitudes, increased empathy for key populations, a reduction in negative and discriminatory moral-based judgements towards key populations and their behaviours, and increased self-perceived capacity to provide appropriate health services to key populations. Over 70% of healthcare workers trained in this programme strongly agreed that this intervention helped to increase awareness of psychosocial vulnerabilities of key populations, and address stigmatising attitudes.

**Conclusion:**

The findings suggest that sensitisation training increases healthcare workers’ knowledge and awareness about specific HIV-related health needs and psychosocial vulnerabilities of key populations, reduces moralising and judgemental attitudes, and results in healthcare workers feeling more skilled to provide appropriate and sensitive services.

## Introduction

Globally, ‘key population’ groups such as sex workers, men who have sex with men (MSM) and people who use drugs (PWUD) are at increased risk of HIV infection compared with the general population.^1,2^ Each year, a significant number of new infections occur amongst these groups, influenced, in part, by the stigmatisation and discrimination experienced in healthcare settings, including refusal of care, patient neglect, provision of differential treatment based on HIV status, breaches of confidentiality, isolation and verbal abuse by staff.^2,3,4,5,6^ Such experiences negatively affect the ability of key populations to engage with both prevention and treatment services, and can lead to delayed treatment, negative effects on retention in care and, ultimately, to poor health outcomes or death.^4,6,7,8^ In addition to experiences of discrimination, few facilities provide the full range of services (e.g. evidence-based substance use disorder services) and equipment (e.g. clean injecting equipment, condoms and compatible lubricants) that might be required by key populations.^9,10,11,12^

In the South African context, MSM, sex workers and PWUD report frequent exclusion from society and high levels of stigma and discrimination in the healthcare setting.^6,8,9,13,14^ Judgemental and moralising views towards sex work, sex between people of the same sex, homosexuality and drug use are frequently expressed within South African communities, and more specifically by individuals working in the healthcare setting, warranting specific attention and address.^6,8,15,16^ Public sector healthcare providers receive scant professional training to support key populations, as highlighted by a review of undergraduate training at one of South Africa’s medical schools.^17^ As such, healthcare workers lack the appropriate skills or knowledge necessary to be adequately equipped to provide these much needed services.^5,6,18^

In response, South African advocates, service providers and researchers have identified the need for increased healthcare worker awareness of the issues affecting key populations, particularly in the context of HIV, and the need to build capacity to provide evidence-based, competent and appropriate health services.^6,17,19,20^ In the light of discrimination by service providers being identified as a major barrier to accessing health services, sensitisation training for healthcare workers about key populations has been recommended to reduce stigmatising attitudes and behaviours towards clients, and thus reduce HIV transmission and incidence rates in South Africa.^21^ The South African National Strategic Plan for HIV, TB and STIs (2017–2022) states the objective to:

[*i*]nvest in expanding training and sensitisation programmes to reduce stigma: Programmes [*to*] … protect those affected by HIV against discrimination and violence and to support access to HIV prevention, treatment, care and support. In addition, human rights and ethics training will be provided for healthcare providers … [*and*] will strengthen the Batho Pele principles.^22^

For the purposes of this article, we use the word ‘sensitisation’ to refer to the process of increasing knowledge of an issue to instil empathy, nurture a ‘sensitive’ disposition shaped by increased awareness, and modify negative attitudes and behaviour, with the intention of reducing discrimination and inequality.^23,24^ Sensitisation training can challenge negative beliefs and shift attitudes through providing factual information, enable individuals to engage emotionally and reflect upon and examine their personal attitudes and beliefs, and encourage positive behavioural intentions through role plays and practical exercises.^25,26^ Evidence from a European context indicates that reductions in structural discrimination and homo-negativity require a multilevel intervention approach, one component of which is enabling changes in attitudes and practices amongst individuals, groups and institutions.^27^ Sensitisation-type training have been shown to have the potential for effecting change at each of these levels, shifting attitudes of individual participants directly and positively, and changing normative professional practice amongst health workers.^27^

Sensitisation training for healthcare workers has been shown to be effective in reducing judgemental and discriminatory attitudes towards marginalised groups.^28^ A mixed-method quantitative and qualitative assessment conducted over a two-year period in Kenya found an overall positive effect of sensitisation training for healthcare workers on attitudes and competencies towards serving MSM patients.^29,30^ Evaluations of stigma reduction or sensitisation training programmes for healthcare workers addressing MSM, transgender or hijra and sex worker populations in India and Bangladesh found substantial positive effects of such training; trained healthcare workers were more likely to exhibit positive, kind, respectful and non-judgemental attitudes towards key populations.^28,31^ Local and regional qualitative research evidence on the effects of sensitisation training for healthcare workers comes from interventions focusing on MSM.^24^ Published data on the outcomes of similar sensitisation training addressing the needs of PWUD and sex workers were not available at the time of writing.

Early key population training efforts in South Africa utilised diverse training methodologies to reach healthcare workers with both sensitisation and medical competency training; however, the training typically focused on individual population groups.^1^ In 2013, several organisations collaborated to develop the ‘Integrated Key Populations Sensitivity Training Programme for Healthcare Workers in South Africa’ in partnership with the South African National Department of Health and the South African National AIDS Council to consolidate sensitisation training across population groups and improve training efficiency.

## The training intervention

In 2013, ‘Healthcare Provision for MSM, Sex Workers, and PWUD: An Introductory Manual for Healthcare Workers in South Africa’ was developed.^32,^^2^ The manual was developed with the intention of improving healthcare workers’ knowledge and awareness of health and related issues affecting key populations, and included topics relating to social norms and values; human sexuality and sexual behaviour; the legal and rights context, socio-structural marginalisation and prejudice; and interventions to foster an enabling healthcare environment. Notably, this training programme did not focus on clinical skills for health service provision to key populations (beyond sexual history taking and awareness raising).

The ‘Integrated Key Populations Sensitivity Training Programme for Healthcare Workers in South Africa’ (key populations’ sensitisation training) was a one-day sensitisation training programme for healthcare workers utilising the training manual, in conjunction with a facilitation guide. A total of 405 healthcare workers (inclusive of nurses, counsellors, social workers and managers) received the sensitisation training between October 2013 and July 2014 across five South African provinces: Eastern Cape, Free State, KwaZulu-Natal, Limpopo and the Northern Cape. The training was conducted by facilitators with prior key population experience, who also received a comprehensive pre-intervention training on the specific content of the integrated key population training. Local government and civil society organisations providing services in the provinces where the training was provided, nominated the healthcare workers who participated in the training.

This article presents the findings from a mixed-method study evaluating the outcomes of the key populations’ sensitisation training in selected locations. The evaluation objectives were to (1) assess changes in the perceptions, attitudes and knowledge of healthcare workers regarding HIV-related issues affecting MSM, sex workers and PWUD, and (2) to assess changes in healthcare workers’ attitudes, for example, stigma and discrimination towards MSM, sex workers and PWUD who access health and related services.

## Methods

### Background to the evaluation research

Of the five provinces that received training, two provincial capitals were selected for evaluation: Bloemfontein (Mangaung Metropolitan District, Free State Province), in which 84 healthcare workers received the sensitisation training, and Mafikeng (Ngaka Modiri Molema District Municipality, North-West Province), in which no training intervention was implemented. These locations were selected because they are characteristic of the public health system outside of major South African metropolitan areas (poor infrastructure, under-resourced, operating with inadequate health personnel, and with significant financial and geographic barriers to accessing healthcare),^33,34^ and because implementation and scale up of key population HIV programmes in these areas were lacking at the time the research was completed. The number of locations was limited by the resources available for the evaluation.

Compared to other South Africa provinces, both North-West and the Free State have low population densities, each containing 7% (*N* = 3 856 174) and 5% (*N* = 2 866 678) of the National population, respectively.^3^ The South African National AIDS Council estimated that in 2018 there were 1228 female sex workers in Mangaung and 1753 in the Ngaka Modiri Molema District. MSM estimates for these districts are 3655 and 3779, respectively. HIV prevalence for these districts is estimated to be similar: 53% for female sex workers and 18% for MSM. PWUD estimates have not been developed because of lack of data.^4^ At the time of the research, health services for MSM and sex workers were limited (but have subsequently increased),^19^ no PWUD outreach programmes or harm reduction services existed^35^ and the availability of water-based condom-compatible lubricants was poor.

This study used a combination of qualitative and quantitative methods to evaluate the intervention. We used quantitative pre- and post-training questionnaires, combined with qualitative interviews, to evaluate the changes linked to the integrated key population sensitisation training. Similar methods have been used to assess changes in healthcare workers’ knowledge, attitudes and practices related to training in African contexts, about HIV treatment and also mental health.^36,37^

#### Quantitative component

Healthcare workers who received the intervention were invited to complete anonymous paper-based pre- and post-training assessment questionnaires. The questionnaires assessed previous training and experience working with key populations, knowledge about key populations’ health needs, as well as attitudes, opinions and beliefs pertaining to key populations and their behaviours. All of the completed paper-based questionnaires were entered into an Excel spreadsheet and imported into Stata 14.^5^ Quantitative analysis included comparison of proportions and frequencies, as well as *t* tests for independent proportions to determine if there were significant differences between pre- and post-training responses.^38^

#### Qualitative component

Prior to the training intervention, baseline in-depth interviews (IDIs) were conducted with eight healthcare workers purposively sampled from four government health facilities in Bloemfontein and four in Mafikeng. Three months after the training intervention was implemented in Bloemfontein, follow-up IDIs were conducted with three healthcare workers in Bloemfontein and one in Mafikeng (the variance in sample size at follow-up was because of respondents declining follow-up interviews). Both baseline and follow-up IDIs explored healthcare workers’ narratives of their experience of working with key populations viz. attitudes and views of the behaviour and social vulnerabilities of this group and of their own capacity to provide appropriate and nonjudgmental services to them. In addition, views with regard to the training of healthcare workers in this field were elicited from the interviewees.

Interviews were conducted by a female socio-behavioural interviewer and a male research assistant, both competent in English and local languages, and trained in the specific implementation of the research tools. IDIs were conducted at the health facility of each participant, often in consultation rooms during tea or lunch breaks. IDIs were conducted in a combination of English and local languages, largely Sesotho and Setswana, and were audio-recorded with consent from the interviewees. Debriefing notes were captured after each IDI by the interviewer and research assistant. Audio-recordings were transcribed and translated into English, and transcriptions were quality-checked by the multi-lingual lead interviewer. NVivo 10 software^6^ was used to code and manage qualitative data. Two coders read and coded the transcripts independently, and any discrepancies were resolved through inter-coder consensus. Predetermined themes and an *a priori* coding scheme were developed to structure the analysis, and coded data were reviewed to determine final themes and outcomes. Comparisons were drawn between the baseline and follow-up evaluation activities per facility, and between facilities.

### Ethical consideration

Approval for the research and permission to access the healthcare staff and facilities were granted by the Free State and North-West Provincial Departments of Health. Ethical approval was also granted by the University of Cape Town’s Human Subjects Research Ethics Committee.^7^

## Findings

### Quantitative findings from pre- and post- training questionnaires

Results from the 401 pre- and 405 post-training assessment questionnaires are shown in Table 1. Awareness of the psychosocial vulnerabilities of key populations, such as violence, stigma and lack of access to healthcare, increased between pre- and post-training assessments. For example, awareness of unfair treatment and discrimination towards sex workers, MSM and PWUD by staff at health facilities increased to 88% after the training (*n* = 355), compared to 71% beforehand (*n* = 286). After the training, 75% (*n* = 302) of respondents were aware that sex workers, MSM and PWUD are more likely to be exposed to violence than the general community, compared to 59% (*n* = 235) prior to training. After the training, 83.5% of trainees (*n* = 338) agreed that this training increased their awareness of how stigma affecting sex workers, MSM and PWUD can limit their access to effective healthcare, compared to 69% (*n* = 276) prior to the training. In the post-training assessments, 67% (*n* = 273) of trainees felt that it was important for their health services to be friendly towards and supportive of sex workers, MSM and PWUD, compared to 50% (*n* = 202) prior to the training. In addition, 56% (*n* = 219) of healthcare workers also strongly felt that they would be able to address discrimination against sex workers, MSM and PWUD in their facilities. Self-reported moralising and prejudicial attitudes related to selling sex, using drugs and same-sex sex were also reduced as a result of the training.

**TABLE 1:** Changes in pre- and post-training assessment items.

Item	Pre-training		Post-training		p*
	(*n* = 401)		(*n* = 405)		
	*n*	%	*n*	%
Aware of link between violence and HIV risk amongst key populations	235	58.60	302	74.60	0.03
Aware how stigma affecting key populations limits access to effective healthcare	276	68.80	338	83.50	< 0.001
Aware that unfair treatment and discrimination by healthcare workers towards key populations are barriers to key populations accessing health services	286	71.30	355	87.70	< 0.001
Aware that key populations may not access health services because of fear of judgement by healthcare workers	305	76.10	340	84.00	0.005
Aware that key populations may not access health services because of concerns of being refused services by healthcare workers	184	45.90	277	68.40	< 0.001
Aware that key populations may not access health services because of concerns of being abused by healthcare workers	202	50.40	273	67.40	< 0.001
Believe that selling sex is immoral or strongly immoral	238	59.40	136	33.60	< 0.001
Believe that using an illegal substance is immoral or strongly immoral	311	77.60	242	59.80	< 0.001
Believe that using an illegal substance is not immoral	90	22.40	163	40.20	< 0.001
Believe that having sex with someone of the same sex is immoral or strongly immoral	229	57.10	130	32.10	< 0.001
Believe that having sex with someone of the same sex is not immoral	172	42.90	275	67.90	< 0.001
Strongly feel comfortable providing health services for SW	121	30.20	160	39.50	0.005
Strongly feel comfortable providing health services for PWUD	101	25.20	156	38.50	< 0.001
Strongly feel comfortable providing health services for MSM	111	27.70	172	42.50	< 0.001

* , Indicates intergroup improvement in post-training compared to pre-training.

### Qualitative findings

In the interviews conducted three months after the training intervention was implemented, there were marked contrasts between opinions expressed by healthcare workers in the intervention group who received training as compared with those in the non-intervention group who did not receive any training. Judgmental views towards key populations, for example, moralising attitudes towards sex work, were voiced by healthcare workers in the non-intervention group:

‘Selling one’s body is not fine … if someone sells their body in town we feel that they are just doing it deliberately. It’s not right … We feel that the person should not be a part of us [*society*] because they sell their body.’ (Non-intervention group)

Prejudicial statements about PWUD were also made by a healthcare worker in the non-intervention group:

‘People who use drugs … after they smoke the drugs they become crazy, they start to steal, they start to harass us and mug us.’ (Non-intervention group)

A healthcare worker from the group who did not receive the training described their own lack of skills and knowledge, and perceived capacity to provide services to sex workers and MSM, and shared the view that training would be beneficial:

‘Men who have sex with men? … I don’t know about those … Sex work is illegal in South Africa … they [*sex workers*] don’t speak [*disclose that they are sex workers to healthcare workers*]. They might come [*to the clinic*] but you can’t know and can’t ask them … you can’t ask them where they work … [*there is a need for training on how to deal with sex workers*].’ (Non-intervention group)

A variety of differences between trained and non-trained healthcare workers later emerged in the follow-up interviews, specifically regarding their judgemental attitudes towards key populations; these are presented below.

Respondents who received the training intervention felt that the training had addressed their previously judgemental attitudes by increasing their knowledge and understanding of various psychosocial and health issues pertaining to key populations. The comment below illustrates a shift away from homo-prejudicial attitudes:

‘Now I can welcome them [*key populations patients*] properly. Because I used to think that they are just naughty before the training. I found out that they are not naughty, at times as a woman you get feelings for other women and as a man you get feelings for other men … I can welcome them because now I know what the problem is. They did not choose … I have learned … not to discriminate them, to end stigma, social stigma.’ (Intervention group, post-training)

Intervention recipients provided further examples of how they perceived the training to have improved their knowledge, specifically in their ability to conduct sexual and risk behaviour history taking. Trained healthcare workers described their perceptions of their own capacity to ask appropriate and relevant questions to key populations patients to provide them with effective care:

‘When a sex worker comes to test [*for HIV*] at the clinic I know what sort of questions to ask.’ (Intervention group, post-training)

Those healthcare workers who received training demonstrated an improved understanding and increased compassion for the challenges, hostility and violence facing key populations in society:

‘These people [*key populations*] feel rejected because we don’t treat them like people.’ (Intervention group, post-training)

‘There are problems, you find that sex workers are beaten and sometimes they are not paid … When they go and report at the police station they tell them off and laugh at them … I have learned a lot about key populations.’ (Intervention group, post-training)

Respondents voiced opinions that the training had enabled them to understand the social marginalisation and discrimination experienced by key populations:

‘I noticed since I went for the [*training*] course … I found that we stigmatise them [*key populations*], we don’t treat them well … A man who has sex with man is ridiculed in society for dating another man, that is stigma … social stigma … I learned a lot from the training.’ (Intervention group, post-training)

Some of the trained healthcare workers explained that their improved understanding and compassion was the result of reflecting on and confronting their own prejudicial attitudes during the training, a necessary step to be able to provide services to key population clients in a sensitive, compassionate, and humane manner:

‘After we went for the training [*we realised*] that … we must treat them [*key populations*] like this [*sensitively*]. When they come here [*to the health facility*] they must feel welcome. They must be like any other patient, we must treat them equally. When a person comes here to share their problems they must not be scared to say that I am a sex worker because they are afraid of how I will react, what I will say to them and if I will judge them. If they say I am a sex worker, I must not even react, I must listen to their story and understand what their problem is … it’s alright because they are also people, we don’t have to isolate them in society, we must treat them like all the other people.’ (Intervention group, post-training)

The way in which the sensitisation training worked to challenge prejudicial views and beliefs, combined with the provision of information about key populations and their risks and vulnerabilities, resulted in self-reported attitude shifts on the part of the healthcare workers, as well as increased introspection on personally held judgemental views:

‘[*After the training*] I also know where I stand … the training opened my eyes so that I could introspect … I noticed that my attitude has changed towards those people [*key populations*] … [*before*] I would see them [*key populations*] but I didn’t understand them. The training opened my eyes and my attitude has changed.’ (Intervention group, post-training)

## Discussion

Findings from this evaluation of South Africa’s first integrated sex worker, MSM and PWUD sensitisation programme for healthcare workers demonstrate that training interventions of this nature can be successful in enabling healthcare workers to better understand the social marginalisation and discrimination experienced by these groups, creating space for them to assess and reflect on engrained social norms that inform discriminatory and judgemental attitudes towards these key populations and their behaviour. Knowledge pertaining to key factors that contribute to poor health outcomes amongst key populations were improved amongst healthcare workers who received the training. Specifically, this evaluation suggests that the sensitisation training improved healthcare workers’ awareness of factors that increase the vulnerability of key populations to HIV infection, including psychosocial issues such as stigma and violence, barriers towards accessing health services, and the consequences of unfair treatment and discrimination by healthcare staff. Findings also indicate that the training intervention resulted in a shift in attitudes, expressed through an increased empathy for key populations, and a reduction in negative and discriminatory moral-based judgements of sex workers, MSM and PWUD and their behaviours. Healthcare workers who received the training also self-reported increased comfort and capability in providing appropriate health services to key populations, suggesting that a sensitisation training of this nature could help to improve the ability of healthcare workers to provide sensitive and appropriate health services to stigmatised and marginalised populations.

The limited data on similar sensitisation-type training programmes that exist support and are congruent with the findings of this study, suggesting that sensitisation training can be efficacious and can facilitate the creation of more tolerant and respectful workplace norms.^25,26,29,30,31^ Outcomes from our study, as previously published data demonstrate, suggest that healthcare workers at both sites held and expressed judgemental and moralising attitudes towards key populations and also lacked the knowledge and awareness of specific key population health needs.^6^ These findings show that healthcare workers who received the training intervention reported an increase in both their support of and acknowledgement of evidence-based interventions for key populations.

## Limitations

There were limitations to this study that should be considered. Firstly, participants in the qualitative component of the study included a small purposively sampled group of healthcare workers, which may have limited the range of data collected. The recruitment and implementation of IDIs were constrained because of the operational logistics of clinics, limited space and the availability of participants because of staff shortages and heavy workloads. In addition, the follow-up IDIs had an even smaller sample size because of respondents declining follow-up IDIs. The participants were not requested to include identifying information on their training questionnaires in order to maximise confidentiality; therefore, pre- and post-training analyses for individuals were not included in this study. Evaluations of future training interventions should include linked pre- and post-intervention assessments to determine individual changes more precisely (e.g. using a unique code) as well as an assessment of key populations service utilisation statistics pre- and post-intervention to identify changes in service uptake. In addition, this study included a three-month period between baseline and follow-up data collection; however, a different follow-up interval may have resulted in different long-term changes in shifts in attitude. Future evaluations would benefit from a larger qualitative sample, and representation from all cadres of healthcare workers.

## Recommendations

Despite the stated limitations, this evaluation suggests that a one-day integrated key population sensitisation training course can positively impact the perceptions, attitudes and knowledge of healthcare workers about HIV-related issues affecting MSM, sex workers and PWUD. Such changes may contribute to the reduction in barriers to access health services and the promotion of welcoming and enabling healthcare facilities. Evidence of this nature is necessary to inform policy recommendations regarding the need for sensitisation training programmes to be integrated into national pre- and in-service training for healthcare workers, and contributes to existing data on outcomes of sensitisation training of healthcare workers, particularly in sub-Saharan Africa where access to sensitised healthcare services for key populations still remains a challenge. Future research is needed to determine how best to utilise refresher training and ongoing mentorship to maintain the longevity of positive attitude changes for periods greater than a year.^26^ Additional operational factors that may improve the efficacy or effectiveness of sensitisation training should also be investigated, including types of content (e.g. clinical competency models), delivery modalities (e.g. online vs. in-person) and participant cadres (e.g. social workers and law enforcement). In addition, future training interventions and evaluations should consider the inclusion of transgender people as a key population, given their vulnerability to HIV infection. Lastly, future research should explore the impact of sensitisation training on key population service utilisation both as a stand-alone intervention and within the context of integrated programming inclusive of community-driven demand creation.

## Conclusion

This evaluation demonstrates that a relatively short (one-day), low-cost training intervention can improve healthcare workers’ levels of knowledge and awareness about the specific HIV-related health needs and psychosocial vulnerabilities of key populations, and reduce levels of prejudice and discrimination.^8^

## Footnotes

1 Examples include MSM Trainings by Anova Health Institute/Health4Men/ICAP South Africa; MSM/Sex Worker/PWUD Trainings by Desmond Tutu HIV Foundation; LGBT Trainings by OUT LGBT Well-Being.

2 Available at: https://www.researchgate.net/publication/261078875_Health_Care_Provision_for_Men_who_have_Sex_with_Men_Sex_Workers_and_People_who_Use_Drugs_-_An_Introductory_Manual_for_Health_Care_Workers_in_South_Africa.

3 StatsSA.

4 South African National AIDS Council Key Populations Cascade Stakeholder Group, February 2018.

5 StataCorp, College Station, TX.

6 NVivo qualitative data analysis Software; QSR International Pty Ltd., Version 10, 2014.

7 Human Subjects Research Ethics Committee Reference number: 390/2013.

8 Building on this and other sensitisation training projects, I-TECH South Africa is developing, implementing and evaluating a comprehensive sensitisation and capacity development pilot intervention programme, with the objective of sensitising clinical and non-clinical staff at the primary care and community levels on issues related to stigma and discrimination, as they relate to five key populations (MSM, people in prisons and other closed settings, sex workers, PWUD and transgender people). https://www.go2itech.org/2017/06/key-populations-sensitization-training-for-health-care-workers-in-south-africa/.
